# Veterinary Register

**Published:** 1901-09

**Authors:** 


### VETERINARY REGISTER.

**[See Editorial, Journal of Comparative Medicine and Veterinary Archives, July, 1901, page 438.]**

### PENNSYLVANIA.

In Pennsylvania all veterinarians before being allowed to practise are required to comply with provisions of the State law. Abstracts as follows:

### AN ACT

To regulate the practice of veterinary medicine and surgery in Pennsylvania.

Section 1. Be it enacted, etc., That every person who shall assume, or use, or cause to be used, any title pertaining to the practice of veterinary medicine or surgery, or any of the branches of veterinary medicine or surgery, shall be a graduate of a legally chartered veterinary college or university, having the power or authority to confer the degree of veterinary surgeon or analogous title, except as provided for in Section 2. And such practitioner shall be required to register in the book kept for that purpose in the office of the prothonotary of the county in which he resides.

Sec. 2. Any person who has assumed the title of veterinary surgeon or analogous title, in this Commonwealth, for the five years preceding the passage of this act, without being entitled to the degree of veterinary surgeon or analogous title, shall be allowed to continue the use of the title; but such person shall appear before the prothonotary of the county in which he resides and make affidavit of that fact; he shall then be recorded as an "existing practitioner."

Sec. 3. The prothonotary shall purchase a book of suitable size, to be known as the "Veterinary Medical Register" of the county, and shall set apart one full page for the registration of each practitioner; and, when any practitioner shall die or remove from the county, the prothonotary shall make a note of the same, and shall perform such other duties as are required by this act.

Sec. 4. Every practitioner who shall be admitted to register shall pay to the prothonotary the sum of one dollar; which sum shall be compensation in full for registration. The prothonotary shall give a receipt for the same, and such registration shall take place within six months from the passage of this act.

Sec. 5. Nothing in this act shall be so construed as to prevent any veterinary surgeon (if legally qualified to use the title) from using the title of "veterinary surgeon" or analogous title in this Commonwealth; but if such veterinary surgeon opens an office or uses the title for the transaction of business he shall be deemed a "sojourner," and shall conform to the requirements of this act.

Sec. 6. Any person who may desire to commence the practice of veterinary surgery or medicine, or any of its branches, in this State, after the passage of this act, and who holds a veterinary diploma, issued, or purporting to have been issued, by any veterinary college or university in this State, another State or foreign country, shall make affidavit before the prothonotary that his diploma has been regularly issued by a legally chartered veterinary college or university, after which such person will be allowed to register as provided for in this act.

SEC. 7. Any person who shall present to a prothonotary a veterinary diploma which has been obtained fraudulently; or which is, in whole or part, a forgery; or shall make affidavit to any false statement, intended to be filed or registered; or shall use the title of veterinary surgeon or analogous title, without conforming to the requirements of this act; or shall otherwise violate or neglect to comply with any of the provisions of this act, shall be deemed guilty of a misdemeanor, and, on conviction, shall be punished for each and every offence, by a fine of one hundred dollars—one-half to be paid to the prosecutor and the other half to be paid to the county; or shall be imprisoned in the county jail of the proper county for a term not exceeding one year, or both or either, at the discretion of the court.

Approved the 11th day of April, A.D. 1889.

JAMES A. BEAVER,
Governor.

### AN ACT

To amend the fourth section of above act as follows:

Every practitioner who shall be admitted to register shall pay to the prothonotary the sum of one dollar, which sum shall be compensation in full for registration. The prothonotary shall give a receipt for the same, and such registration shall take place at any time prior to the first day of January, one thousand eight hundred and ninety-two, but not on or after that day; *Provided*, That nothing in this act shall be taken or construed to apply to persons who practice castration of domestic animals, and no other form of veterinary medicine or surgery.

Approved the 29th day of April, A.D. 1891.

ROBERT E. PATTISON,
Governor.

### AN ACT

To establish a State Board of Veterinary Medical Examiners to regulate the practice of veterinary medicine and surgery in the State of Pennsylvania.

SECTION 1. Be it enacted, etc., That a State Board of Examiners known as the State Board of Veterinary Medical Examiners, is hereby established, to consist of five members, who shall be of good standing in the veterinary profession, shall be graduates of a recognized veterinary college or colleges, and who shall hold office until their successors are appointed and duly qualified. Said board shall have power to adopt by-laws and regulations such as they may deem advisable to carry into effect the provisions of this act.

. . . . . . . . .

SEC. 4. From the fees provided for by this act the board may pay, not to exceed said income, all proper expenses incurred by its provisions, and if any surplus above said expenses shall remain, such examiners shall receive a reasonable remuneration from the said surplus for their work.

SEC. 5. For the purpose of examining applicants for license said board of Veterinary Medical Examiners shall hold two or more stated or special meetings in each year, due notice of which shall be made public at such time and places as they may determine. At said stated or special meetings a majority of the members of the board shall constitute a quorum thereof, but the examination may be conducted by a committee of one or more members duly authorized by said board.

SEC. 6. The said Board of Veterinary Medical Examiners shall examine all diplomas as to their genuineness, and each applicant for a license shall submit to a theoretical and practical examination, said examination to be written, oral or both. Such examinations shall include the following subjects: Veterinary anatomy, surgery, practice of medicine, obstetrics, pathology, chemistry, veterinary diagnosis, materia medica, therapeutics, physiology, zoötechnics, sanitary medicine, and meat-inspection and milk-inspection.

SEC. 7. Said board shall issue, forthwith, to each applicant who has passed such examination successfully, and who shall have been adjudged to be duly qualified for the practice of veterinary medicine and surgery, a license to practice the same in the State of Pennsylvania. Such license, issued pursuant to this act, shall be subscribed by the officers of the Board of Veterinary Medical Examiners. It also shall have affixed to it, by the person authorized to affix the same, the seal of this Commonwealth. Before said license shall be issued it shall be recorded in a book to be kept in the office which said board shall establish, for the purpose of carrying out the provisions of this act; and the number of this book and the page therein containing said recorded copy shall be noted upon the face of said license. Such records shall be open to public inspection with proper restrictions as to their preservation.

SEC. 8. From and after the first Monday in September, one thousand eight hundred and ninety-five, any person not heretofore authorized to practice veterinary medicine and surgery in this State, and desiring to enter upon such practice, may deliver to the secretary of the veterinary medical board, upon the payment of a fee of ten ($10) dollars, a written application for license, together with satisfactory proof that the applicant is more than twenty-one years of age, is of good moral character, has obtained a competent common school education, and has received a diploma conferring the degree of veterinary medicine from some legally incorporated veterinary college of the United States, or a diploma or license conferring the full right to practice all the branches of veterinary surgery in some foreign country; applicants who have received their degree in veterinary medicine after the first day of July, one thousand eight hundred and ninety-six, must have pursued the study of veterinary medicine for at least three years, including three regular courses of lectures of at least six months each in different years, in some legally incorporated veterinary college or colleges, prior to the granting of said diploma or foreign license. Such proof shall be made, if required, upon affidavit. Upon making the said payment and exhibiting the before-named proof, the examining board, if satisfied with the same, shall issue to such applicant an order for examination. In case of failure at any such examination, the candidate, after the expiration of six months and within two years, shall have the privilege of a second examination by the same board to which application was first made, without the payment of an additional fee; and it is further provided that the applicants examined and licensed by State Boards of Veterinary Medical Examiners of other States, on payment of a fee of 10 ($10) dollars to the examining board, and on filing in the office of said board a copy of said license, certified by the affidavit of the president or secretary of the board of such other State, showing also that the standard of examinations and other requirements adopted by that State Board of Veterinary Medical Examiners is substantially the same as that provided for by this act, shall without further examination, receive a license conferring upon the holder thereof all the rights and privileges provided by sections eight and nine of this act.

Sec. 9. From and after the first Monday in September, one thousand eight hundred and ninety-five, no person shall enter upon the practice of veterinary medicine and surgery in the State of Pennsylvania unless he has complied with the provisions of this act, and shall have exhibited to the prothonotary of the court of common pleas of the county in which he desires to practice veterinary medicine and surgery, a license duly granted to him as hereinbefore provided; whereupon he shall be entitled, upon the payment of one ($1) dollar, to be duly registered in the office of the prothonotary of the Court of Common Pleas in the said county; and any person violating any of the provisions of this act shall be guilty of a misdemeanor, and upon conviction thereof in the court of quarter sessions of the county wherein the offence shall have been committed, shall pay a fine of not more than two hundred dollars ($200) for each offence; said examining boards shall be the prosecutor in all cases.

Sec. 10. Nothing in this act shall be construed to interfere with or punish commissioned veterinarians in the United States Army, or any lawfully qualified veterinarians residing in other States or countries meeting registered veterinarians of this State in consultation, or any veterinarian residing on the border of a neighboring State and duly authorized under the laws thereof to practice veterinary medicine and surgery therein whose practice extends into the limits of this State; *Provided*, That such practitioner shall not open an office or appoint a place to meet patients or receive calls within the limits of Pennsylvania. And nothing in this act shall be construed to prohibit the practice of veterinary medicine and surgery within this Commonwealth by any practitioner who shall have been duly registered before the first Monday in September, one thousand eight hundred and ninety-five, and one such registry shall be sufficient warrant to practice veterinary medicine and surgery in any county in this Commonwealth. Nothing in this act shall apply to persons who castrate domestic animals or to persons gratuitously treating diseased animals.

Sec. 11. All acts or parts of acts of Assembly inconsistent herewith shall be and are hereby repealed.

Approved the 16th day of May, A.D. 1895.

Daniel H. Hastings,
Governor.

The foregoing is a true and correct copy of the act of the General Assembly, No. 55.

Frank Reeder,
Secretary of the Commonwealth.

The examinations under these acts are held on the third Monday and Tuesday in June and December.

The Board of Examiners consists of—President, Dr. James A. Sallade, Auburn, Pa.; Secretary-Treasurer, Dr. W. Horace Hoskins, 3452 Ludlow Street, Philadelphia, Pa.; Dr. Jacob Helmer, 311 Spruce Street, Scranton, Pa.; Dr. W. H. Ridge, Trevose, Pa.; Dr. J. C. McNeil, 26 Fourth Street, Pittsburg, Pa.

**[In the following list the names and data printed without comment are those furnished to us by postal card with the signature of the individual.**

**Those with an asterisk [*] are those to whom we have sent, by United States mail, an envelope with prepaid postage and a request for return if not delivered in ten days; as the envelopes have not been returned, we presume that the letter has been delivered, that the individual in question exists, but that he has neglected to return the enclosed postal card with the information requested.—ED. JOURNAL.]**

### PENNSYLVANIA.

| *Name.* | *Street.* | *Town.* | *College, Graduated.* | *County, Registered.* |
|---|---|---|---|---|
| Adams, John W. | 25 S. 34th st | Philadelphia | V. D. Univ. Pa., 1892 | Philadelphia, 1895 |
| Aikens, Alex. | 2119 Vine st | Philadelphia | * | ...... |
| Ainsworth, T. W. | B. of A. I. | Pittsburg | * | ...... |
| Albright, John | ...... | Calcium | * | ...... |
| Alexander, C. | ...... | Coburn | ........... | Centre, 1890 |
| Alexander, H. M. | ...... | Marietta | V. D. Univ. Pa., 1876 | Lancaster, 1881 |
| Alexander, Joseph | ...... | Coraopolis | * | ...... |
| Aley, John W. | ...... | Sheffield | * | ...... |
| Allebach, J. M. | ...... | Skippack | * | ...... |
| Allen, Francis S. | 800 N. 17th st | Philadelphia | American Vet., 1884 | Philadelphia |
| Allis, N. H. | ...... | Wyalusing | * | ...... |
| Ament, Jacob | ...... | Harold | * | ...... |
| Ames, Arthur | ...... | Garrwood | * | ...... |
| Ames, Arthur, Jr. | ...... | Saltsburg | * | ...... |
| Anderson, A. S., Sr. | ...... | Reeds Gap | * | ...... |
| Anderson, J. W. | ...... | Edinboro | * | ...... |
| Andrews, W. F. | 575 Green st | Meadville | Chicago Vet., 1894 | Crawford, 1894 |
| Ankeny, A. J. | ...... | Edie, | * | ...... |
| Ankeny, Calvin H. | Meyers st | Confluence | ........... | Somerset, 1889 |
| Appel, W. W. | ...... | Pleasant Val. | N. Y. C. V. S., 1889 | Bucks, 1889 |
| Applebee, J. F. | ...... | Wesleyville | * | ...... |
| Ardary, R. W. | 31st & Liberty | Pittsburg | * | ...... |
| Arthur, Wm. | ...... | Tamaqua | * | ...... |
| Ash, David | ...... | Chandler's Valley | * | ...... |
| Ash, Wesley | 608 N. Main st | Meadville | ........... | Crawford, 1889 |
| Ash, William | ...... | Parkersford | * | ...... |
| Atkinson, J. A. | Maine st | Goodwill Hill | R. C. V. S., 1865 | Warren, 1890 |
| Ayers, E. N. | ...... | Garland | * | ...... |
| Bachman, B. F. | Bayard st Schenley Rdg. Acad. | Pittsburg | V. D. Univ. Pa., 1888 | Lancaster |
| Bachman, L. J. | 514 S. Broadway | Bethlehem | * | ...... |
| Bachman, M. L. | 14 S. Rubel av | Bethlehem | * | ...... |

| *Name.* | *Street,* | *Town.* | *College, Graduated.* | *County, Registered.* |
|---|---|---|---|---|
| Bagnall, Chas. H. | Kings st | Chambersb'g | Chicago Vet., 1892 | Elk and Mercer, 1892, 1894 |
| Bagnall, Wm. P. | 224 Hickey st | Warren | Chicago Vet. | Warren, 1891 |
| Bailey, C. M. | ...... | Le Roysville | * | ...... |
| Bair, Samuel T. | ...... | Green Bank | * | ...... |
| Baker, H. W. | ...... | Dixonville | * | ...... |
| Baldwin, Daniel E. | ...... | Somerset | * | ...... |
| Barber, David L. | ...... | Taylorville | * | ...... |
| Barber, Raymond | ...... | Newtown | * | ...... |
| Barclay, F. J. | ...... | Industry | ...... | Beaver |
| Barker, F. P. | Railroad st | Coburn | | Centre, 1890 |
| Barndt, Aaron H. | ...... | Salfordville | * | ...... |
| Barr, George A. | ...... | Decker's P't | * | ...... |
| Barr, John A. | ...... | Utah | * | ...... |
| Barr, Samuel S. | ...... | Decker's P't | * | ...... |
| Bartholomew, Henry | ...... | Nittany | * | ...... |
| Bartholomew, J. C. | ...... | Berwyn | * | ...... |
| Bartilson, B. A. | ...... | W. Middle'n | * | ...... |
| Bartilson, J. M. | ...... | W. Middle'n | * | ...... |
| Bartley, W. J. | 23 W. 12th st | Erie | * | ...... |
| Bartman, Jacob | ...... | Salfordville | * | ...... |
| Bassler, Daniel F. | ...... | Woodbury | ...... | Bedford, 1891 |
| Baum, A. L. | ...... | Telford | * | ...... |
| Baum, Christian | ...... | Hamburg | * | ...... |
| Baum, Howard L. | ...... | Trumbauersville | Ontario Vet., 1892 | Bucks and Montgomery, 1892 |
| Baum, Thomas A. | ...... | Shelly Sta. | ...... | Bucks, 1889 |
| Bausman, A. B. | ...... | Millersville | ...... | Lancaster, 1889 |
| Bausmann, John, Jr. | ...... | Millersville | * | ...... |
| Beach, Jesse E. | 119 Jared st | Du Bois | ...... | Clearfield, 1890 |
| Beachy, Lee | ...... | Elk Lick | * | ...... |
| Beachy, R. M. | ...... | Elk Lick | * | ...... |
| Beale, W. M. | ...... | Walnut | * | ...... |
| Bear, Harry H. | ...... | Mt. Joy | * | ...... |
| Beaver, James A. | ...... | Waynesboro | * | ...... |
| Beaver, Joseph | ...... | Carrolltown | * | ...... |
| Bechard, Thomas | ...... | Honey Brook | * | ...... |
| Beck, W. | ...... | Langhorne | * | ...... |
| Becker, Jacob N. | 13 College st | Palmyra | ...... | Lebanon, 1889 |
| Beecher, Wm. H. | ...... | Manheim | * | ...... |
| Behney, Cyrus E. | ...... | Lebanon | * | ...... |
| Beinhauer, J. R. | ...... | Pittsburg | * | ...... |
| Bender, W. K. | ...... | Lititz | * | ...... |
| Benner, Willis G. | ...... | Doylestown | Ontario Vet., 1890 | Bucks, 1890 |
| Bennett, John | ...... | Breadysville | * | ...... |
| Bennett, N. C. | ...... | Stephensville | * | ...... |
| Berrett, E. A. | ...... | N. Jackson | * | ...... |
| Beshore, Peter | ...... | Myerstown | * | ...... |
| Best, John A. | ...... | Stone Church | * | ...... |
| Bethune, J. G. | Front st | Punxsut'ney | Ohio Vet., 1893 | Jefferson, 1893 |
| Bickel, Daniel G. | ...... | Pleasant Run | * | ...... |
| Bickel, S. D. | 118 E. Main st | Norristown | * | ...... |
| Bickley, John S. | 1533 Christian | Philadelphia | * | ...... |
| Bicknell, G. W. | 1403 Webster av | Pittsburg | * | ...... |
| Bieber, U. S. Grant | ...... | Kutztown | * | ...... |
| Biehl, Joel | ...... | Moselem Spgs. | * | ...... |
| Biehl, John K. | ...... | Molltown | ...... | Berks, 1889 |
| Biehl, Samuel K. | 841 Locust st | Reading | * | ...... |
| Biehl, Walter G. | ...... | Loyalsockville | Amer. Vet., 1898 | Lycoming, 1899 |
| Biehm, Oscar, Jr. | ...... | Coopersburg | * | ...... |
| Bigelow, J. V., | ...... | Monongahela | * | ...... |
| Biggs, Basil | Cor. High and Washington | Gettysburg | ...... | Adams, 1889 |
| Biggs, Wm. M. | Cor. High and Washington | Gettysburg | ...... | Adams, 1889 |
| Bilger, Enos | ...... | Globe Mills | * | ...... |

| *Name.* | *Street.* | *Town.* | *College, Graduated.* | *County, Registered.* |
|---|---|---|---|---|
| Bilger, Isaac | ...... | Kratzerville | * | ...... |
| Bilger, Jesse | ...... | Middleburg | * | ...... |
| Bilger, Joel | ...... | Middleburg | * | ...... |
| Bilger, John | 24 S. Allegheny | Bellefonte | ...... | Centre, 1898 |
| Bilger, W. D. | ...... | Mt. Pleasant Mills | * | ...... |
| Birch, Wm. A. | 1513 Frankford | Philadelphia | * | ...... |
| Bishop, S. P. | ...... | Carlisle | * | ...... |
| Bitner, George W. | ...... | Up. Strasburg | * | ...... |
| Bitner, Samuel A. | ...... | Potter's Mills | * | ...... |
| Bittles, Edmund E. | 119 E. North st | New Castle | Ontario Vet., 1890 | Erie, Mercer, 1890 Lawrence, 1895 |
| Black, Alvin F. | ...... | Turnip Hole | * | ...... |
| Blaker, George C. | ...... | Richborough | Amer. Vet., 1887 | Bucks, 1887 |
| Blakeslee, R. P. | ...... | Spartansburg | * | ...... |
| Bland, Charles | 6448 Ridge av | Philadelphia | Ontario Vet., 1888 | Philadelphia, 1889 |
| Blank, G. G. | 115 N. 9th st | Allentown | Ontario Vet., 1884 | Lehigh, 1889 |
| Blank, H. L. | ...... | Lehighton | * | ...... |
| Blatt, Jonathan | ...... | Centreport | * | ...... |
| Blatt, William B. | ...... | Centreport | * | ...... |
| Bloom, Lewis I. | ...... | Curwensville | * | ...... |
| Boaker, Jay C. | ...... | Falls Creek | * | ...... |
| Boman, M. G. | ...... | Westfield | * | ... .. |
| Bombay, Sylvester | ...... | Salem T'n'p | * | ...... |
| Bomboy, John | ...... | Hetherville | * | ...... |
| Bond, Ira L. | ...... | Tamaqua | * | ...... |
| Bonner, James C. | ...... | Garland | * | ...... |
| Booker, S. F. | ...... | Kittanning | * | ...... |
| Boone, T. W. | ...... | Hazelton | * | ...... |
| Boorem, John | ...... | Paradise Val. | * | ...... |
| Borneman, H. G. | ...... | Boyertown | * | ...... |
| Bortner, Henry M. | ...... | Codorus | * | ...... |
| Bortner, Josiah D. | ...... | Codorus | * | ...... |
| Bostock, C. Baxter | Hilltop Farm | Montg. Sq. | Edinburg, Lon., 1868 | Delaware and Montgomery, 1889 |
| Bowen, Aloy B. | Water st | Everett | ...... | Bedford, 1890 |
| Bower, Henry | ...... | Collegeville | * | ... .. |
| Bowers, W. J. | ...... | Round Top | * | ...... |
| Bowersox, D. F. | ...... | Aaronsburg | ...... | Centre, 1889 |
| Bowersox, John | ...... | Coburn | * | ..... |
| Bowersox, Jos. | ...... | Middleburg | * | ...... |
| Bowles, H. | ...... | Pittsburg | * | ..... |
| Bowser, Samuel J. | ...... | Somerset | * | ...... |
| Boyd, C. W. | 26 S. Diamond | Allegheny | * | ...... |
| Brackbill, John C. | ...... | E. Lampeter | * | ...... |
| Brackbill, Marsh L. | ...... | Strasburg | V. D. Univ. Pa., 1895 | Lancaster, 1895 |
| Bradley, John L. | ...... | Mercersburg | * | ...... |
| Bradley, Orin C. | ...... | Franklin | * | ...... |
| Brake, G. Bruce | ...... | Everett | Ontario Vet., 1890 | Bedford, 1898 Franklin, 1890 |
| Brallier, G. W. | ...... | Berlin | * | ...... |
| Brandt, Jacob | ... .. | Franklin | * | ...... |
| Brandt, John W. | ...... | York | * | ...... |
| Brechbill, Geo. L. | ...... | Pleas't Unity | * | ...... |
| Breidenthal, W. H, | ...... | Loysburg | * | ...... |
| Brendle, J, H. | ...... | Red Run | * | ...... |
| Brenneman, H. F. | ...... | Mount Joy | * | ...... |
| Brensinger, David | ...... | Grill | * | ...... |
| Bridge, Chas. E. | 228 N. 53d st | Philadelphia | * | ...... |
| Bridge, Francis | 228 N. 53d st | Philadelphia | * | Philadelphia, 1889 |
| Bridge, John | 1012 W. 2d st | Chester | * | ...... |
| Briggs, Chas. A. | ...... | Factoryville | * | ...... |
| Brigham, Geo. W. | ...... | Mansfield | * | ...... |
| Brindle, D. C. | ...... | Scotland | * | ...... |
| Brink, Simeon L. | ...... | Wayne | * | ...... |
| Britenstine, M. S. | ...... | Lebanon | * | ...... |
| Britton, E. G. | ...... | Guy's Mills | * | ...... |

| *Name.* | *Street.* | *Town.* | *College, Graduated.* | *County, Registered.* |
|---|---|---|---|---|
| Britton, Benj. | ...... | E. Brunswick | * | ...... |
| Brodhead, C. W. | Cherry st | Montrose | ...... | Susquehanna, 1889 |
| Broadhead, W. M. | ...... | Media | * | ...... |
| Brossman, C. W. | High st | Womelsdorf | Ontario Vet., 1891 | Berks and Lebanon, 1891 |
| Brown, D. C. | ...... | Piolett | * | ...... |
| Brown, George E. | ...... | Rural Valley | * | ...... |
| Brown, John F. | ...... | Airville | * | ...... |
| Brown, Sherred | Eddison st | Eldred | * | ...... |
| Brubaker, Edwin S. | ...... | Durlach | * | ...... |
| Brubaker, H. W. | ...... | Berlin | * | ...... |
| Brubaker, John K. | ...... | Rohrerstown | * | ...... |
| Brumgard, G. W. | ...... | Two Taverns | * | ...... |
| Brumgard, John | ...... | Hanover | * | ...... |
| Bryan, Milton M. | ...... | Altoona | * | ...... |
| Bryce, John | 5th & French | Erie | Ontario Vet., 1870 | Erie, 1889 |
| Bryer, John | ...... | Beaver Falls | * | ...... |
| Buchanan, John W. | ...... | Liverpool | * | ...... |
| Buckham, James | Eleventh st | Beaver Falls | Ontario Vet., 1889 | Beaver, 1894 |
| Buckwalter, D. | ...... | E. Lampeter | * | ...... |
| Buell, Charles A. | ...... | Blooming Val. | * | ...... |
| Buish, A. F. | 248 S. 22d st | Philadelphia | * | ...... |
| Burkey, George | ...... | Milledgeville | * | ...... |
| Burkhart, Christian | ...... | Frankford T'n'p | * | ...... |
| Burkholder, A. F. | ...... | Big Spring | * | ...... |
| Burkholder, D. W. | ...... | W. Pennsboro | * | ...... |
| Burnell, Charles G. | ...... | Philadelphia | * | ...... |
| Burns, Caston | ...... | Elmwood City | * | ...... |
| Burt, James H. | 1115 Peach st | Erie | Ontario Vet., 1895 | Erie, 1897 |
| Burwell, Edgar | 123 Lehigh st | S. E. Easton | * | ...... |
| Busch, Joseph | ...... | Kashner | * | ...... |
| Bush, I. M. | Bishop st | Bellefonte | Chicago Vet., 1888 | Centre and Adams, 1888 |
| Bush, William E. | ...... | Damascus | * | ...... |
| Bussier, Wm. | Island road near 79th st | Philadelphia | * | ...... |
| Butler, Burdine | ...... | Howard | * | ...... |
| Butler, H. L. | ...... | Gravity | * | ...... |
| Butterfield, J. F. | ...... | S. Montrose | Ontario Vet., 1886 | Susq'hanna, 1889 |
| Butts, Charles | ...... | Sabinsville | * | ...... |
| Byers, George | ...... | Snowden | * | ...... |

(*To be continued.*)

